# Reciprocal associations between early maladaptive schemas and depression in adolescence: long-term effects of childhood abuse and neglect

**DOI:** 10.1186/s13034-023-00682-z

**Published:** 2023-11-30

**Authors:** Yumeng Wang, Yemiao Gao, Jinmeng Liu, Rong Bai, Xia Liu

**Affiliations:** Institute of Developmental Psychology, Faculty of Psychology, Beijing Normal University, No. 19 Xinjiekouwai Street, Beijing, 100875 China

**Keywords:** Depression, Early maladaptive schemas, Childhood abuse and neglect, Early adolescence

## Abstract

**Background:**

Adolescent depression has grown to be a major social concern in China. During the coronavirus pandemic, the incidence of depression among Chinese adolescents increased substantially. More research is required to inform the prevention and intervention of adolescent depression in China. Depression is associated with Early Maladaptive Schemas (EMSs). Childhood abuse and neglect are distal antecedents of adolescent depression. It is not known how depression and EMSs interact in adolescence and how childhood abuse and neglect contribute to this relationship. This study aimed to examine the reciprocal relationships between depression and EMSs, as well as the long-term effects of childhood abuse and neglect on depression and EMSs during adolescence. The work also investigates gender differences in these mechanisms.

**Methods:**

Using a two-wave longitudinal design, we recruited 3,485 Chinese adolescents (*M*_age_ = 13.2; 43.2% females) from three Shanxi Province, China middle schools. All participants completed self-report questionnaires addressing childhood abuse and neglect, depression, and EMSs. Structural equation models examined reciprocal relationships between depression and EMS, as well as the effect of childhood abuse and neglect on depression and EMSs. Multi-group analysis addressed gender differences.

**Results:**

Results indicated that greater depression predicted more EMSs measured later, but EMSs did not predict subsequent depression. Childhood abuse and neglect had different effects on depression and EMSs during adolescence. Specifically, exposure to childhood abuse related to more severe depression and EMSs in adolescence and contributed to the perpetuation of EMSs by increasing depression. Exposure to childhood neglect showed a direct effect on depression and indirectly reinforced subsequent EMSs through depression. There were no gender differences.

**Conclusion:**

These findings contribute to a better understanding of the emergence and course of depression in early adolescence, suggesting that childhood abuse and neglect are critical early risk factors. Additionally, depression plays a key role in promoting schema perpetuation among adolescents exposed to childhood maltreatment, providing important implications for relevant prevention and intervention in early adolescence.

## Introduction

Recently, adolescent depression has become a major health concern in China. A meta-analysis found the prevalence of depression among Chinese adolescents was 24.3% [1]. During the coronavirus pandemic 2019 (COVID-19) lockdown in China, the incidence of adolescent depression increased significantly [2]. Adolescent-onset depression more strongly relates to adverse adult outcomes than does depression onset in other periods [3]. This suggests a need for more research on adolescent depression in China to explore the emergence and course of depression to inform effective prevention and intervention programs. According to models of cognitive therapy, cognitive vulnerability is a key element in understanding the emergence of depression [4]. Recent work addressing the deep structure of cognitive vulnerability focused on early maladaptive schemas (EMS). Several studies demonstrated that EMSs predict depression in adolescents [5]. However, this relationship could also work in reverse. Specifically, EMSs form and may be fluid during adolescence [6]. Experiencing depression might reinforce adolescents’ EMSs and causes EMSs to develop into stable psychopathological mechanisms [6, 7]. This can lead to more severe functional impairments in adulthood [8]. Therefore, it is necessary to conduct further research to examine reciprocal relationships between depression and EMSs.

Childhood abuse and neglect are crucial early causes of both depression and EMSs [9, 10]. However, the underlying mechanisms linking childhood abuse and neglect to depression and EMSs in adolescence remain poorly understood. According to the dimensional model of adversity and psychopathology (DAMP), exposure to childhood abuse and neglect influences children’s neurodevelopmental processes in different manners, leading to distinct developmental consequences [11]. Few studies explored the unique effects of childhood abuse and neglect on depression and EMSs simultaneously. The current study focuses on reciprocal associations between EMSs and depression in adolescence and the effects of childhood abuse and neglect in this process. The work also explores whether these mechanisms vary by gender.

### Early maladaptive schemas and adolescent depression

Early maladaptive schemas (EMSs) refer to a range of broad, diffuse, and dysfunctional cognitions about the self, the world, and relationships with others. These schemas develop during childhood and adolescence [12]. According to schema therapy, EMSs are key psychopathological foundation for mental disorders, including depression [12]. Several empirical studies have supported that EMSs are risk factors for worsening depression. For example, in a longitudinal study with a sample of university students in Canada, endorsing more severe EMSs (e.g., abuse, defectiveness, and failure) predicted greater depression over time [13]. Consistent with findings in adults, these same relationships existed in studies conducted with adolescents [14–16].

The scar hypothesis proposes that people are more likely to develop negative beliefs after suffering depression [17, 18]. This suggests a bidirectional relationship between depression and EMSs. However, few studies examine this bidirectional relationship and those have found inconsistent conclusions. For example, Gómez-Odriozola and Calvete [19] found a bidirectional relationship between depression and EMSs in a sample of Spanish adolescents. EMSs (disconnection and rejection) predicted depression six months later, and depression led to a subsequent strengthening of EMSs. In contrast, LaGrange et al. [7] found only a unidirectional effect of depression on maladaptive cognitive schema, a construct parallel to EMSs. This work did not find that maladaptive schema leads to depression. Given these inconsistencies, more research is needed to clarify relationships between EMSs and adolescent depression over time. Additionally, relationships between depression and cognitive vulnerability in adolescents differ between Chinese and Western cultures [20, 21]. As such, it is important to explore the relationships between depression and EMSs among Chinese adolescents. This study aimed to examine the reciprocal relationships between EMSs and depression over time in a sample of Chinese adolescents.

### The effects of childhood abuse and neglect

Childhood maltreatment is a robust contributor to depression and EMSs in adolescence [9, 10]. However, research exploring the underlying mechanisms of relationships among these three factors is limited. Most work focused on the mediating role of EMSs in the childhood maltreatment and depression relationship [14, 22]. In the context of childhood maltreatment, depression may be an important mechanism in the construction of EMSs. Specifically, childhood maltreatment impairs the ability to cope with negative emotions and puts adolescents at elevated risk for depression [23, 24]. This may cause adolescents to hold negative self-perceptions and interpret events negatively [25, 26], thus reinforcing their EMSs. This suggests a dynamic association among childhood maltreatment, depression, and EMSs over time. Recently, evidence from a longitudinal study found interactions between negative life events, depression, and EMSs over time during adolescence [27]. However, this study only focused on recent negative life events. The long-term dynamic effects of childhood maltreatment on depression and EMSs in adolescence remain unclear. Understanding how childhood maltreatment contributes to the development of depression and EMSs during adolescence may have implications for inventions to prevent depression and subsequent psychopathology deterioration among adolescents exposed to childhood maltreatment.

Most studies either examined childhood maltreatment as a general category [28] or the cumulative risk of childhood maltreatment [29]. However, these approaches do not account for the unique effects of different types of childhood maltreatment [11]. According to the dimensional model of adversity and psychopathology (DAMP), childhood abuse and childhood neglect are the two core underlying dimensions of childhood maltreatment [30]. Exposure to childhood abuse is the threat dimension. The threat dimension refers to experiences of harm or threat of harm. Exposure to childhood neglect is the deprivation dimension. This refers to the absence of species-expected cognitive, physical, or social inputs [31]. Within DAMP, these two dimensions co-occur and increase the risk for psychopathology but have unique effects on developmental consequences [11, 32]. Empirically, a small number of studies examined the effects of childhood abuse and neglect on EMSs [33] or the effects of these two types of childhood maltreatment on depression [34]. However, empirical research on the dynamic relationship among childhood abuse and neglect, depression, and EMSs is still scarce. Therefore, it is necessary to divide childhood maltreatment into abuse and neglect, and to explore the long-term dynamic effects of childhood abuse and neglect on EMSs and depression.

### Gender differences

The divergence between boys and girls in psychopathology risk emerges and tends to increase during adolescence [35, 36]. Therefore, it is necessary to explore gender differences in psychopathological mechanisms. Gender differences do exist in both depression and EMSs among adolescents. Girls reported more severe depression [37] and had higher EMSs scores [6]. Gender differences in susceptibility to childhood abuse and neglect also exist. Specifically, the effect of childhood abuse and neglect may be larger for girls [38]. For instance, several studies demonstrated that among adolescents exposed to childhood abuse and neglect, girls are more likely to suffer from mental health problems such as depression [39], psychotic symptoms [40], and non-suicidal self-injury [41]. Although prior work established gender differences in childhood abuse and neglect, depression, and EMSs, there remains a gap in understanding the gender differences in mechanisms connecting childhood abuse and neglect to depression and EMSs in adolescence. Such information is essential for developing more targeted preventive interventions.

### The current study

This study examined reciprocal associations between EMSs and depression in adolescence and the effects of childhood abuse and neglect on this process. There were two aims. The first was to examine reciprocal associations between EMSs and depression using a cross-lagged model. We predicted that more EMSs at T1 relate to more depression at T2 and that more depression at T1 also related to more EMSs at T2 (Hypothesis 1). The second aim was to examine the effects of childhood abuse and neglect on EMSs and depression by including childhood abuse and neglect in the cross-lagged model. We hypothesized that more childhood abuse (Hypothesis 2) and neglect (Hypothesis 3) related to more EMSs and depression at T1 and T2. Additionally, childhood abuse (Hypothesis 4) and neglect (Hypothesis 5) indirectly predict adolescents’ depression at T2 via their EMSs at T1 and indirectly predict EMSs at T2 via depression at T1. Considering the potential developmental differences between boys and girls in adolescence, we also examined variations in these mechanisms by gender.

## Method

### Participants and procedure

Data came from two assessments 6 months apart (Time 1: Dec 2021 and Time 2: Jun 2022) from an ongoing longitudinal study in China. We randomly contacted three middle schools in Shanxi Province, China, introduced them to the study purpose and asked about their willingness to cooperate. All three schools gave permission. Subsequently, we recruited a total of 3925 students from 69 classes at T1. All were in the seventh or eighth grade. Due to attrition at T2, only 3829 students participated. We used two criteria to exclude invalid questionnaires. The first exclusion criterion was time to completion. We excluded those participants who completed the questionnaire in less than 50% of the median time [42]. The second exclusion criterion was the failure to correctly answer attention check questions (e.g., Please respond with “Totally Agree” for this item). The final sample included 3485 participants, 1505 girls and 1980 boys, aged between 10 and 17 years, with most (98.8%) aged between 12 and 15 years, the mean age was 13.2 years (*SD* = 0.9).

With the cooperation of the schools, we conducted online data collection in the computer room of the schools during regular school hours. Before collecting data, the researcher emphasized to participants that they were free to withdraw at any time during the research, that all their responses were confidential, and that the data was only used for scientific research. During the formal collection, participants clicked on the web-link of the questionnaire and completed self-report questionnaires following standardized instructions. Most students completed the questionnaire within 35 min. After the research, each participant received stationery as a gift for participation. This study was approved by Beijing Normal University’s Ethics Review Committee. The written informed consent had been obtained from all participants and their caregivers.

### Measures

#### Childhood abuse and childhood neglect (T1)

The translated version of the Childhood Trauma Questionnaire-Short Form (CTQ-SF) [43] measured childhood abuse and childhood neglect. The original scale consists of 28 items and has five subscales, namely physical abuse, emotional abuse, physical neglect, emotional neglect, and sexual abuse. We adopted physical abuse and emotional abuse subscales to address childhood abuse, with a total of 10 items, and used physical neglect and emotional neglect subscales to address childhood neglect, with a total of 10 items. Participants reported their history of abuse and neglect before the age of 12 (e.g., “Before I was 12 years old, someone in my family beat me and left me with bruises or scars on my skin.”). Responses to items are on a five-point Likert scale, ranging from 1 (*never*) to 5 (*always*). Higher scores indicated more severe abuse and neglect in childhood. The Chinese version of CTQ-SF shows adequate reliability and validity among Chinese adolescents [44]. In the present study, the Cronbach’s αs for childhood abuse and childhood neglect were 0.70 and 0.76 at T1 respectively.

#### Early maladaptive schemas (T1 & T2)

The translated version of the Schema Questionnaire for Children (SQC) [45] addressed early maladaptive schemas (EMSs). The scale consists of 15 items. Sample items include “People I love will never be there for me”. Every item corresponds to a specific type of EMSs [12]. Responses to items are on a 6-point Likert scale ranging from 1 (*completely disagree*) to 6 (*completely agree)*. Higher scores indicate higher levels of EMSs. The SQC shows adequate reliability and validity [45–47] as well as applying to adolescents aged 11 to 16 [48]. The Chinese version of SQC has been translated following the standard translation/back-translation procedure. Then a pretest among Chinese adolescents (*n* = 237) showed that the translated scale had adequate reliability (Cronbach’s α = 0.79) and validity (CFI = 0.929, TLI = 0.907, RMSEA = 0.050, SRMR = 0.058). In the present study, Cronbach’s αs were 0.85 and 0.88 at T1 and T2.

#### Depression (T1 & T2)

The 10-item Centre for Epidemiological Studies Depression Scale (CES-D-10) [49, 50] addressed depression. Each item on the scale contains a core identifying question for depressive symptoms [51]. Sample items include “I felt that everything I did was an effort”. Participants reported how often they had experienced symptoms in the past week. Responses range from 1 (*rarely or never [less than one day]*) to 4 (*most of the time [5 to 7 days]*). Higher scores indicate more severe depressive symptoms. According to criteria used in previous studies [52, 53], we adopted a cutoff score of 20 or higher for significant depression symptoms. The Chinese version of the scale has been validated to have adequate reliability and validity in Chinese adolescents [54–56], and Cronbach’s αs were 0.83 and 0.84 at T1 and T2 in the present study.

#### Demographic characteristics (T1)

Demographic questions assessed age, gender, and subjective socioeconomic status. The gender was coded as 1 (*male*) and 0 (*female*). The MacArthur Scale addressed subjective socioeconomic status [57]. For this scale, participants indicate their status on a 10-step ladder. Responses range from 1 (*lowest living standards*) to 10 (*highest living standards*). The MacArthur Scale has adequate reliability and validity among Chinese adolescents [58].

### Data analytic strategy

First, the preliminary data analyses were conducted, and Pearson correlation analysis examined the bivariate associations between all study variables. Welch’s t-test examined the gender differences of the main variables [59]. Then, a cross-lagged model of EMSs and depression addressed Hypothesis 1, which was estimated using structural equations model. To examine Hypotheses 2 and 3, both childhood abuse and neglect were added as predictors in the cross-lagged model in a structural equations model (final model) that included pathways of childhood abuse and neglect to EMSs and depression at both times. Finally, multi-group analysis addressed whether the paths differed across gender. We modeled childhood abuse and neglect, EMSs at both times and depression at both times as latent variables. In models, subjective socioeconomic status and age served as control variables.

The preliminary analyses utilized SPSS 23.0 (Version 23.0; IBM Corp, 1989–2015). Mplus version 8.3 estimated cross-lagged models and the subsequent final model [60]. To control for measurement error and improve the parsimony of the models, we employed an items-to-construct balancing technique to develop parcels as manifest indicators of the latent constructs [61, 62]. Mediation analyses in the final model used maximum likelihood (ML) estimation and bootstrapping with 5000 bootstrapped samples. The root mean square error of approximation (RMSEA), comparative fit index (CFI), Tucker-Lewis index (TLI), and standardized root mean square residual (SRMR) addressed fit.

## Results

### Description statistics and correlations

A total of 19.7% (*n* = 687) adolescents showed depression at T1, and 23.2% (*n* = 807) adolescents showed depression at T2. Table 1 presents correlations, means, and standard deviations for the main study variables. The bivariate correlations among all the main study variables were statistically significant. Specifically, childhood abuse and neglect were positively associated with the EMS and depression at both T1 and T2, showing small to large correlation coefficients (*r* = .23 ~ .50, *p* < .01) [63]. Early maladaptive schema was positively correlated with depression at both T1 and T2 and between T1 and T2, showing medium to large correlation coefficients (*r* = .46 ~ .64, *p* < .01).

**Table 1:** Correlations, means, and standard deviations among main study variables

Variable	1	2	3	4	5	6
1 Childhood abuse	1					
2 Childhood neglect	0.45^**^	1				
3 EMSs (T1)	0.43^**^	0.31^**^	1			
4 EMSs (T2)	0.32^**^	0.23^**^	0.50^**^	1		
5 Depression (T1)	0.50^**^	0.41^**^	0.61^**^	0.47^**^	1	
6 Depression (T2)	0.37^**^	0.34^**^	0.46^**^	0.61^**^	0.64^**^	1
*M*	12.5	12.6	36.6	36.0	15.7	16.2
*SD*	3.5	3.9	11.1	12.2	5.3	5.5
Range	10 − 46	10 − 38	15 − 90	15 − 90	10 − 40	10 − 40

*Note*: EMSs = Early Maladaptive Schemas. Range: the minimum and maximum scores achieved in sample. ^*^p < .05. ^**^p < .01. ^***^p < 0. 001

In addition, we compared the main study variables across gender groups (boys: *n* = 1980, 56.8%; girls: *n* = 1505, 43.2%). For both childhood abuse (*t*(2902.14) = 3.80; *p* < .001, *Cohen’s d* = 0.14) and neglect (*t*(2993.16) = 2.07; *p* < .05, *Cohen’s d* = 0.08), girls reported weakly higher scores than boys. Moreover, compared with boys, girls also had higher levels of early maladaptive schema (T1: *t*(3136.22) = 5.48; *p* < .001, *Cohen’s d* = 0.20; T2: *t*(3209.58) = 6.30; *p* < .001, *Cohen’s d* = 0.21) and depression(T1: *t*(2917.26) = 6.47; *p* < .001, *Cohen’s d* = 0.24; T2: *t*(2964.28) = 6.58; *p* < .001, *Cohen’s d* = 0.24) at both T1 and T2, with small to medium effect size.

### Reciprocal associations between early maladaptive schemas and depression

**Fig. 1:**
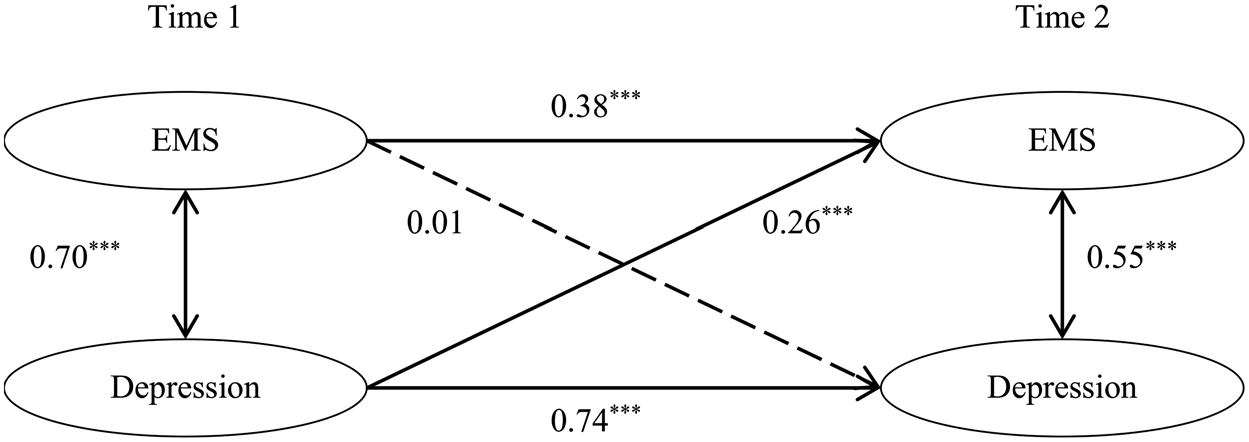
The reciprocal associations between early maladaptive schemas and depression across two waves. Path coefficients are standardized estimates. The model controlled for subjective socioeconomic status and age. EMSs = Early Maladaptive Schemas. *p < .05, **p < .01, ***p < .001

The cross-lagged analysis investigated reciprocal associations between EMSs and depression from T1 to T2. The latent variable cross-lagged model fit the data well, CFI = 0.980, TLI = 0.972, RMSEA = 0.049, SRMR = 0.018. As shown in Fig. 1, autoregressive paths for both EMSs and depression were positive and significant across time (*p* < .001). T1 depression was positively associated with T2 EMSs (*β* = 0.26, *p* < .001). However, the predictive effect of T1 EMSs on T2 depression was not significant (*β* = 0.01, *p* = .63). The model accounted for 35.5% of the variance in T2 EMSs and 55.8% in T2 depression.

### Effects of childhood abuse and neglect on early maladaptive schemas and depression

To investigate the effect of childhood abuse and neglect on EMSs and depression in adolescence, we added both childhood abuse and neglect to the cross-lagged model as predictors. The model fit the data well, CFI = 0.976, TLI = 0.968, RMSEA = 0.043, SRMR = 0.029.

**Fig. 2:**
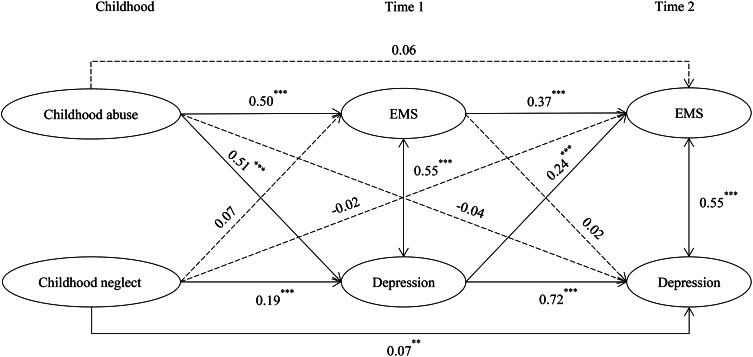
The effects of childhood abuse and neglect on early maladaptive schemas and depression across two waves. Path coefficients are standardized estimates. The model controlled for subjective socioeconomic status and age. EMSs = Early Maladaptive Schemas. *p < .05, **p < .01, ***p < .001

As shown in Fig. 2, more experiences of childhood abuse related to increases in T1 EMSs (*β* = 0.50, *p* < .001) and T1 depression (*β* = 0.51, *p* < .001). The indirect path from childhood abuse on T2 EMSs via T1 depression was significant (indirect effect = 0.12, Boot SE = 0.02, Boot 95%CI = [0.08, 0.17]), indicating that T1 depression mediated the effect of childhood abuse on T2 EMSs. More childhood neglect related to increases in T1 depression (*β* = 0.19, *p* < .001). The indirect path from childhood neglect on T2 EMSs via T1 depression was significant (indirect effect = 0.05, Boot SE = 0.01, Boot 95%CI = [0.03, 0.07]), indicating that T1 depression mediated the effect of childhood neglect on T2 EMSs. Greater childhood neglect related to more depression at T2 (*β* = 0.07, *p* < .01). All direct and indirect effects are displayed in Table 2. The model accounted for 35.1% of the variance in T2 EMSs and 55.7% in T2 depression.

**Table 2:** Standardized effects and bootstrapped 95% confidence intervals in the final model

Pathways	*β*	*SE*	*Boot 95%CI*
Childhood abuse → T1 EMS	**0.50** ^***^	0.03	0.441, 0.564
Childhood abuse → T1 Depression	**0.51** ^***^	0.03	0.455, 0.579
Childhood abuse → T2 EMS	0.06	0.04	−0.013, 0.126
Childhood abuse → T2 Depression	−0.04	0.03	−0.102, 0.024
Childhood abuse → T1 Depression → T2 EMS	**0.12** ^***^	0.02	0.084, 0.165
Childhood abuse → T1 EMS → T2 Depression	0.01	0.01	−0.018, 0.038
Childhood neglect → T1 EMS	0.07	0.03	−0.004, 0.128
Childhood neglect → T1 Depression	**0.19** ^***^	0.03	0.125, 0.255
Childhood neglect → T2 EMS	−0.02	0.03	−0.069, 0.036
Childhood neglect → T2 Depression	**0.07** ^**^	0.03	0.021, 0.121
Childhood neglect → T1 Depression → T2 EMS	**0.05** ^***^	0.01	0.027, 0.068
Childhood neglect → T1 EMS → T2 Depression	0.001	0.002	−0.002, 0.007

*Note*: EMSs = Early Maladaptive Schemas. *p < .05. **p < .01. ***p < 0. 001

### Gender differences

Multi-group analysis addressed whether the path coefficients in the final model differed across gender. Specifically, we compared the baseline model (no constraints) and the constrained model to test gender differences. The baseline model allowed structural paths to vary. The constrained model set structural paths as equal across gender. There was not a significant difference between the baseline model [χ2(246) = 1130.725, CFI = 0.971, RMSEA = 0.045, SRMR = 0.035] and the constrained model [χ2(258) = 1144.448, CFI = 0.971, RMSEA = 0.044, SRMR = 0.035], *Δχ*^*2*^ (12, *N* = 3485) = 13.72, *p* = .32, indicated that the final model paths did not differ by gender.

## Discussion

Given the significant impact of adolescents’ depression on the development of psychopathology throughout life, a better understanding of the emergence and pernicious course of depression in adolescence is essential for informing effective prevention and intervention strategies. In the current study, we explored the reciprocal relationships between depression and EMSs in adolescents and the long-term effect of childhood abuse and neglect on this process. We also considered gender differences. The result revealed that depression predicted later EMSs, but EMSs did not predict depression. Additionally, childhood abuse predicted both depression and EMS directly, while childhood neglect predicted depression. Additionally, depression mediated the effect of childhood abuse and neglect on EMSs. We found no gender differences in these processes.

Findings revealed a unidirectional relationship between depression and EMSs. Higher levels of depression predicted more severe EMSs later, which is consistent with the work of LaGrange et al. [7]. According to the scar hypothesis, being in a depressed state can increase adolescents’ cognitive vulnerability [17]. This process makes them more susceptible to irrational beliefs, changes their expectations, and leads to a tendency to think negatively [17, 18], thus aggravating EMSs. Inconsistent with previous work [14, 19], EMSs were not related to subsequent depression. This may reflect that EMSs are not fully established during early adolescence [6]. Most work demonstrating that EMSs predicted to depression were based on adolescents in mid to late adolescence, whereas the present study focused on early adolescence. Although researchers assume EMSs originate early in childhood [45], the stability and dysfunction of EMSs may not become evident until mid to late adolescence [64, 65]. This suggests the effect of EMSs on depression in early adolescence is relatively limited.

Our study is the first to explore the long-term dynamic effect of childhood abuse and neglect on depression and EMSs in Chinese adolescents. As hypothesized, results showed that exposure to childhood abuse directly predicted more severe depression and EMSs. According to the framework of the dimensional model of adversity and psychopathology (DAMP), early experiences of abuse (threat) related to long-term alterations in neurocircuitry associated with emotion reactivity [66]. These alternations may magnify emotional responses to negative emotional cues, increasing the risk of subsequent depression [67, 68]. Additionally, exposure to abuse relates to children’s feelings of insecurity, mistrust, and poor self-worth, which leaves them with negative beliefs or expectations that they are shameful, others are unreliable and that the world is unpredictable [12]. Over time, these beliefs can evolve into EMSs [22].

We also found that childhood abuse indirectly affects EMSs via the mediating effect of depression, suggesting a detailed process of schema perpetuation [12]. Specifically, childhood abuse can worsen adolescent depression by impairing emotion regulation [24]. This, in turn, may induce more negative beliefs that aggravate EMSs [18]. These results indicated that exposure to childhood abuse puts adolescents at risk for depression and EMSs and may contribute to the persistence of EMSs indirectly. This suggests that more attention should be given to preventing depression in adolescents with a history of childhood abuse to safeguard physical and mental health.

Regarding childhood neglect, we found that childhood neglect had a direct and long-lasting effect on the severity of adolescent depression. This consistent with the work of Wang et al. [34]. And within the DAMP, exposure to childhood neglect (deprivation) relates to severe impairment of executive functioning [69] and language ability [70]. These impairments lead to deficits in social problem-solving and social-emotional learning [34, 67], contributing to the persistence of depression.

As with the findings regarding childhood abuse, childhood neglect had an indirect effect on EMSs via prior depression. Previous research showed that childhood neglect relates to alterations in reward-related neural circuitry, especially blunted ventral striatum reactivity [71]. Ventral striatum reactivity is the pathophysiology of typical depressive symptoms like anhedonia [72]. These depressive symptoms, in turn, make it difficult for adolescents to have positive experiences or hold a positive view of themselves or others, thus exacerbating EMSs.

Notably, Consistent with the work of Kindt et al. [73], we did not find a mediating role of EMSs. In the present study, although childhood abuse predicted more severe EMSs in adolescence, EMSs did not lead to a subsequent strengthening of depression, suggesting that EMSs, as negative cognitive styles, may not be an important mechanism of childhood abuse and neglect causing early adolescence depression. Based on the current results, intervention programs targeting negative cognitive styles like EMSs may not have a satisfactory effect on early adolescent depression. Previous work has also found that intervention strategies targeting negative cognitive styles, despite their frequent application, showed only small effects in preventing adolescent depression [74, 75]. This suggests that more effective measures should be considered in clinical practices for the prevention and treatment of depression in early adolescence. For example, Kindt et al. [73] pointed out that interventions aimed at reducing dependent negative life events (e.g., projects to train social skills, combat school bullying, and reduce parent-child conflicts) might be more effective in reducing depression in early adolescents than interventions aimed only at changing negative cognitive styles. Further prevention and intervention of early adolescent depression may benefit from considering the particular negative life events in adolescents and making targeted clinical decisions.

Several limitations should be noted. First, this study measured EMSs in general, while different subtypes of EMSs may correspond to different psychopathology in adolescents [6]. Future studies should identify subtypes of EMSs that are particularly relevant to depression, allowing for a deeper understanding of the development of depression and EMSs during adolescence. Second, we selected a sample of general adolescents and did not address clinical. Considering that clinical populations endorse more extreme EMSs [76], and the underlying psychopathological mechanisms of depression may differ in clinical and non-clinical samples [22, 77]. The conclusions in this study do not necessarily generalize clinical groups. Further work should employ both clinically referred and non-referred samples of adolescents to address differences between those groups. Third, the present study’s focus is on early adolescence. Future work should investigate depression and EMSs with a more comprehensive sample of adolescence to provide a clearer picture of the development of depression and cognitive vulnerability. Additionally, although we selected three middle schools in two cities for sampling, all the participants were located in Shanxi, a province in northern China, which limited the representativeness of the sample. We should be cautious when generalizing the current research findings to the adolescent population in other regions. More studies are warranted to explore the long-term dynamic relationship among EMSs, depression, childhood abuse and neglect in diverse regional samples.

Despite these limitations, the findings of this study enable a better understanding of the emergence and course of depression in Chinese adolescents and have implications for future practice. Our research highlighted the long-term impacts of experiencing abuse on children’s emotional and cognitive development, showing that childhood abuse increases the risk of depression and EMSs and may indirectly contribute to the worsening of EMSs by aggravating depression. Although most Chinese parents recognize scientific parenting practices, some families still engage in certain forms of child abuse such as shouting and slapping [78]. In this study, the incidence of childhood abuse was 24.0%. Just like the Chinese proverb, “Beating is caring and scolding is loving”, in traditional Chinese parenting, harsh discipline embodies parental care and responsibility [79, 80]. As a result, some Chinese parents adopt physical or verbal punishment when their children do something wrong out of concern [81]. However, exposure to abuse contributes to later increases in depression and EMSs. This suggests a need for effective parental skill training to reduce child abuse. Additionally, parents and educators should pay more attention to adolescents with a history of childhood abuse. Supporting these adolescents may prevent the emergence of depression and EMSs.

Childhood neglect is a “silent” form of maltreatment that appears to have small or insignificant effects on adolescent mental health [82, 83]. However, our results found that neglect showed a longer lasting effect on adolescent depression than abuse. Additionally, neglect indirectly reinforces EMSs. Chinese culture does not recognize neglect as a form of maltreatment in a way [84]. Traditional Chinese parenting tends to place more emphasis on children’s academic, social, and moral achievements than on their emotional needs [85]. This may lead to neglect, especially emotional neglect. Given this, it is crucial to emphasize the negative effects of neglect on children’s mental health and to advise parents in prioritizing their children’s emotional and physical needs in order to protect them from early neglect. In addition, our findings also emphasized the vulnerability of girls to EMSs and depression compared with boys, as well as the fact that girls are more likely to suffer from childhood abuse and neglect. This suggests the need to further promote gender equality, pay more attention to girls’ mental health and develop gender-friendly measures in the schools and communities.

## Conclusion

This study provides new evidence regarding the relationship between depression and EMS in early adolescence and highlights the distal effects of childhood abuse and neglect on this process. Results suggest that severe depression relates to more endorsement of EMSs later in life and that depression mediates the effects of both childhood abuse and neglect on EMSs in adolescence. Using a longitudinal design, our findings addressed the emergence and development of depression and EMSs during early adolescence. Future studies should explore the dynamic relationship between emotional and cognitive vulnerability throughout adolescence. In practice, these findings also provide important insight for adolescent depression prevention programs, adding fuel to the ongoing studies to explore more effective targeting points for preventing early adolescent depression.

## Data Availability

The datasets support the findings of the current study are available from the corresponding author upon reasonable request.

## Abbreviations

EMS: Early Maladaptive Schemas
DAMP: Dimensional Model of Adversity and Psychopathology
CTQ-SF: Childhood Trauma Questionnaire-Short Form
SQC: Schema Questionnaire for Children
CES-D-10: Centre for Epidemiological Studies Depression Scale 10-item measure
